# Dr. Marshall H. Webb

**Published:** 1883-02

**Authors:** 


*Dr. Marshall H. Webb.*—A bibliographical notice of Dr Webb. prepared by Dr. Longnecker, his former pupil and friend appears in the February number of the *Dental Cosmos*, but the occasion demands something more than a passing tribute to Dr. Webb's disinterested and valuable services to the profession. He gave time and effort without stint to teach all who chose to learn whatever improved methods he had devised, and it is safe to say that all over the country better dentistry is being done to day in many offices as a result of Dr. Webb's clinics and teaching. A sad side of the case is that, with the feeling that life was before him, years of labor, he gave more of his life and strength for the general good than he could afford to ; more doubtless than he would have done had he foreseen the early termination of his career. He has left a widow and three children unprovided for. His record is finished and is before the profession. Already, without solicitation, a score of operators have expressed their sense of obligation and their sympathy by generous subscriptions to a testimonial fund. The editor of the *Dental Cosmos* has been solicited to act as treasurer, and will gladly receive and acknowledge any subscriptions which may be sent as a token of appreciation of the unselfish work of Dr. Webb and of sympathy with his bereaved family.

Dr. J. W. White, the editor of the *Dental Cosmos,* has requested the editor of this JOURNAL to bring the matter before the profession in this section, and receive and forward subscriptions, which he will gladly do, and appeals to the profession of Baltimore city and State of Maryland to respond to this meritorious action. Any subscriptions can be forwarded directly to Dr. J. W. White, S. S. White Dental Manufacturing Co. Cor. Chestnut and 12th Streets, Philadelphia, or to the editor of this JOURNAL. through Messrs. Snowden & Cowman, Publishers, 86 West Fayette Street, Baltimore.

